# Challenges in Aphid Pest Management Including Global Case Studies and Prospects

**DOI:** 10.3390/plants15152392

**Published:** 2026-08-05

**Authors:** Adalbert Balog, Artúr Botond Csorba, Hugh D. Loxdale, Sándor Keszthelyi, Adriana Aurori, Ciprian George Fora

**Affiliations:** 1 Department of Horticulture, Faculty of Technical and Human Sciences, Sapientia Hungarian University of Transylvania, Aleea Sighișoarei 2, 540485 Târgu Mureș, Romania; 2 Department of Agrobiology, Institute of Biology, Faculty of Sciences, University of Pécs, Ifjúság Str. 6, 7624 Pécs, Hungary; 3 School of Biosciences, Cardiff University, The Sir Martin Evans Building, Museum Avenue, Cardiff CF10 3AX, UK; loxdale@web.de; 4 Department of Agronomy, Institute of Agronomy, Kaposvár Campus, Hungarian University of Agriculture and Life Sciences, S. Guba Str. 40, 7400 Kaposvár, Hungary; keszthelyi.sandor@uni-mate.hu; 5 Faculty of Horticulture and Business for Rural Development, University of Agricultural Sciences and Veterinary Medicine, Str. Manastur Nr. 3-5, 400372 Cluj-Napoca, Romania; adriana.aurori@usamvcluj.ro; 6 Faculty of Engineering and Applied Technologies, University of Life Sciences “King Michael I” from Timișoara, Calea Aradului 119, 300645 Timișoara, Romania

**Keywords:** biological control, climate change, emerging resistance, non-target species, policy implications, virus vectors

## Abstract

Aphids are among the most economically important pests affecting agricultural productivity worldwide. Despite various control strategies, managing aphid populations remains a challenge due to their rapid reproduction, ability to transmit pathogenic plant viruses, and their resistance to insecticides. The present article presents the current major difficulties experienced in the control of aphid pests, detailing various case studies, whilst recent scientific findings from Europe, the United States of America, and other global regions are described in order to clarify the challenges and the possibilities in sustainable pest management. Overall, we conclude that the complexity of aphid pest control demands integrated management strategies supported by robust policy frameworks. Enhanced collaboration between researchers, policymakers, farmers, and industry stakeholders is essential to develop sustainable solutions that address both immediate pest control needs and long-term agroecosystem resilience. Furthermore, studies are necessary, which especially include molecular genetics and ecological assessments, in order to understand the life history and rapid evolution of aphids, and hence, formulate the most rational means to combat them, both in the greenhouse and field.

## 1. Introduction

Aphids (Hemiptera: Aphididae) represent a large family of soft-bodied and plant-parasitic insects which feed on the phloem sap of their hosts and act as efficient vectors of predominantly host-specific pathogenic plant viruses [1]. Of some 5000 aphid species described globally, around 250 are considered serious pests of agriculture, horticulture and forestry [2]. These insects cause substantial damage to a wide variety of crops, including wheat, maize, soybean, as well as many horticultural and forestry species.

Global estimates of yield losses due to their damage can reach 50% in certain crops [3]. The rapid reproductive capacity and genetic plasticity of aphid populations have enabled them to quickly adapt to changing environmental conditions and insecticide-selective pressures, as extensively documented, especially in the peach potato aphid, *Myzus persicae*, and melon-cotton aphid, *Aphis gossypii*, populations worldwide [4]. Moreover, the role of aphids as vectors of a broad spectrum of plant viruses exacerbates crop losses and complicates pest management, as many viruses are transmitted rapidly and before insecticide treatments can take effect [1,5,6]. Emerging resistance to biological insecticides and microbial biocontrol agents also poses an additional challenge [3,7], emphasizing the need for diversified control tactics, continuous research, and resistance management, even within biocontrol frameworks [8].

Furthermore, research aimed at combating these pests must focus on the development of innovative control methods, including biotechnological approaches such as RNA interference and host plant resistance, alongside improved resistance monitoring and climate-adaptive strategies [9,10]. In this light, it is timely to present the current major difficulties experienced in the control of aphid pests. Here detailed case studies, including recent scientific findings from Europe, the USA, and other important crop-growing global regions worldwide, are described in order to clarify the challenges and the possibilities in sustainable pest management.

Unlike previous reviews that primarily summarize individual management strategies or aphid biology, the present review integrates recent evidence on insecticide resistance, biological control constraints, aphid-transmitted viruses, climate-driven changes in aphid ecology, predictive modelling, and emerging resistance to biological insecticides within a single conceptual framework. Particular emphasis is placed on understanding the interacting factors responsible for current management failures and identifying future priorities for sustainable aphid management under changing environmental and agricultural conditions.

## 2. Biological Characteristics Underlying Aphid Pest Success

Of insect pests, aphids are critically important due to their enormous reproductive potential, which includes cyclical parthenogenesis, a life cycle alternating between asexual (parthenogenetic) and sexual reproduction [11,12]. During favorable environmental conditions, particularly in spring and summer, aphid populations primarily reproduce via viviparous parthenogenesis, wherein largely genetically identical daughters are produced without mating [13,14]. This results in exponential population growth within a very short time [15]. One female can produce up to 80–100 offspring, with a generation time as short as 7–10 days under optimal temperature conditions. This rapid reproduction is facilitated by telescoping of generations, whereby embryonic development begins within the developing embryos of their mothers, effectively allowing for three generations to overlap simultaneously [16]. Such a strategy accelerates colonization and makes early pest detection crucial to effective management. The phenotypic plasticity of aphid genotypes, particularly their capacity for polyphenism, is likely a crucial factor underlying their adaptive potential [17]. In *M. persicae*, one of the few apparently truly polyphagous aphid species [18], this remarkable plasticity appears to be at least partly driven by gene duplications. This species is capable of exploiting a broad host range, feeding on over 100 plant species across more than 40 botanical families [19].

In colder seasons, many aphid species switch to a sexual phase to produce cold hardy overwintering eggs, enhancing survival in temperate climates. However, in greenhouse environments and warmer regions, anholocyclic (obligate parthenogenetic) populations persist throughout the year, creating continual pest pressure and enabling quick selection for adaptive traits, including insecticide resistance [20]. Genetic studies have revealed significant clonal diversity among aphid populations. For example, studies on *M. persicae* populations across Europe (France) identified a high frequency of “superclones” with broad host range, elevated fitness, and resistance traits [21]. In Canada and the USA, invasive asexual lineages (‘clones’) of the soybean aphid, *Aphis glycines*, have been tracked using polymorphic microsatellite markers, highlighting rapid geographic spread and evolution in response to host plant resistance and management regimes [22]. In the pea aphid, *Acyrthosiphon pisum*, certain obligate parthenogenetic (OP) lineages harbour a recessive allele that entirely suppresses the production of sexual females. These lineages reproduce exclusively through asexual females all year-round, yet still give rise to a single generation of males in the autumn [23]. Such OP males are capable of transmitting the recessive allele to other lineages during mating, facilitating the recurrent formation of highly fecund OP genotypes that may be preadapted to a variety of local environmental conditions [23]. The domestication of leguminous crops may have substantially influenced the evolutionary diversification of *A. pisum*. This species represents a complex of at least 15 sympatric biotypes, each highly specialized on one or a limited number of host plants within the family Fabaceae [24]. In a comparable case, *M. persicae* comprises a distinct sub-specific population adapted to cultivated tobacco, *Nicotiana tabacum*, which has become widely established throughout Europe [25].

Moreover, aphid adaptability is helped by symbiotic relationships with endosymbiotic bacteria such as the obligate symbiont *Buchnera aphidicola*, which provides essential amino acids, as well as facultative (secondary) symbionts like *Hamiltonella defensa* that confer resistance to hymenopterous parasitoid wasps [26,27,28,29,30]. These symbionts can be horizontally transferred and selected for under biotic stress, further complicating aphid management. The presence of the obligate symbiont *B. aphidicola* significantly influences multiple aspects of aphid biology. For instance, empirical studies have demonstrated that these symbionts play a crucial role in supporting aphid growth, development, and the production of viviparous offspring [31,32]. Moreover, in *A. gossypii*, exposure to elevated temperatures has been shown to enhance fecundity, while apparently exerting no significant effect on the abundance of the obligate symbiont *B. aphidicola*. This pattern stands in contrast to that observed in the black bean aphid, *Aphis fabae*, where heat stress leads to high mortality, likely due to a substantial reduction in *Buchnera* populations [32]. Similarly, in both the blue alfalfa aphid, *Acyrthosiphon kondoi*, and the pea aphid, *A. pisum*, heat exposure results in a marked decline in *B. aphidicola* abundance, accompanied by reduced survival, slower development, and decreased fecundity [32]. Secondary symbionts are frequently shared among divergent aphid lineages and appear to be transmitted both vertically and horizontally across matrilines [33]. A number of previous studies have indicated that these symbiotic bacterial communities contribute to the expression of various biologically relevant traits in aphids, including, as aforementioned, resistance to hymenopterous parasitoids, as well as increased tolerance to heat stress and shifts in host plant utilization [34].

Recent advances in genomic tools have enabled whole-genome sequencing of key aphid pests, uncovering gene duplication events in detoxification pathways and expansion of gene families involved in chemosensory reception [35]. These genetic innovations contribute to host plant switching and resistance evolution, posing major challenges to conventional pest control strategies. Thus, the combination of rapid asexual reproduction, clonal variability, symbiont-mediated defenses, and genomic plasticity makes aphids exceptionally resilient agricultural pests, necessitating specific adaptive management approaches.

## 3. Development of Insecticide Resistance

The intensive application of synthetic insecticides has led to the development of multiple resistance mechanisms in aphid populations, making chemical control increasingly ineffective. Resistance to major classes of insecticides, including organophosphates, carbamates, pyrethroids, and neonicotinoids, has been widely documented, e.g., [36].

In *M. persicae*, mutations in the voltage-gated sodium channel gene result in knockdown resistance (*kdr*) and super-*kdr* phenotypes, which confer reduced sensitivity to pyrethroids. Similarly, point mutations in the acetylcholinesterase gene (*ace1*) are associated with resistance to carbamates and organophosphates [36]. These mutations are now widespread in aphid populations across Europe and Asia. Another major resistance mechanism is enhanced metabolic detoxification. Overexpression of cytochrome P450 monooxygenases (particularly *CYP6CY3*), esterases, and glutathione S-transferases has been observed in several aphid species. For instance, *CYP6CY3* has been linked to neonicotinoid resistance in *M. persicae*, and its copy number variation contributes to dose-dependent resistance [4,37]. Metabolic resistance is especially problematic because it can confer cross-resistance to multiple chemical classes [36]. Reduced cuticular penetration is a third mechanism, whereby modifications in the insect cuticle limit insecticide absorption. In aphids, this resistance is typically seen in combination with metabolic resistance, compounding the challenge of effective control [38].

Behavioral resistance, which is, however, less understood, also plays a significant role. Changes in feeding behaviour, such as reduced probing or avoidance of treated surfaces, have been reported [39]. Globally, aphid resistance has had serious agronomic consequences. In the United States, *A. gossypii* has developed in Californian cotton high levels of resistance to neonicotinoids and organophosphates, prompting shifts toward seed treatments and rotation with other modes of action [40]. In the UK, resistant clones of *M. persicae* have rendered insecticides largely ineffective [41].

Compounding the issue is the flow of resistance genes between aphid populations via sexual recombination or migration. Resistance management is further hampered by insufficient implementation of insecticide rotation and monitoring programmes. In many regions, lack of resistance diagnostics and overreliance on a narrow spectrum of products have accelerated the emergence of resistant populations [42]. Integrated resistance management (IRM) strategies are therefore crucial. These include the use of action thresholds, rotating insecticides with different modes of action, incorporating biocontrol agents, and developing resistant plant varieties. However, the adoption of such strategies remains limited in smallholder systems due to technical, financial, and informational constraints.

## 4. Historical and Contemporary Economic Impacts of Aphid Pests

In the global pest *M. persicae*, resistance to organophosphate insecticides was first noted in peach orchards in the USA in the mid-1950s [4]. Thereafter, such resistance was noted extensively in the field, including against organophosphates, carbamates and organochloride insecticides [4]. The evolution of insecticide resistance in these chemical classes, due to intense selection, in turn a result of overzealous usage including prophylactic strategies, has led to efforts to reduce the input of both chemical types, whilst at the same time other insecticides have been sought [7,43]. It was already well known that natural pyrethroid, extracted from chrysanthemums, was toxic to insects, having a knockdown action on the insect nervous system. Investigations, especially by Michael Elliott (1924–2007) and coworkers at Rothamsted in Harpenden, Hertfordshire, UK, were able to eventually synthesize a series of synthetic pyrethroids based on the core molecular structure of the natural compound, which proved to have considerably more potency than this [44,45]. Besides their significant knockdown action against flying insects, including aphids [4], pyrethroids persist for a relatively short time in the field (half-life in soil ranges from 1 day to 16 weeks), being broken down by bacteria and sunlight, but are nevertheless toxic to mammals and birds, and especially to fishes and non-target invertebrates [46].

To date, around 20 major aphid pests have evolved insecticide resistance to one or more insecticides since the introduction of these chemicals globally [7,47]. A brief list of some of the major pest species involved and the control strategies directed at them is given below (Table 1), showing the trends in aphid-related costs and losses over time.

The economic impact of aphid outbreaks has been well documented across several cropping systems. Following its introduction into the USA in 1986, the Russian wheat aphid, *Diuraphis noxia*, caused substantial economic losses in wheat and barley production, with annual direct yield losses peaking at approximately US$274 million in 1988 before declining to less than US$10 million by 1993 as monitoring and management strategies improved [48]. Similarly, outbreaks of the sugarcane aphid, *Melanaphis sacchari*, in sorghum in South Texas have resulted in considerable regional economic losses, estimated at US$ 38.8 million in 2014, including approximately US$23.3 million in farm profit losses together with increased pesticide and management costs [49]. Long-term studies in the UK have demonstrated the considerable economic importance of cereal aphids, particularly through their role as vectors of Barley yellow dwarf virus (BYDV) [50]. Yield losses are often driven more by virus transmission than by direct feeding, and severe epidemics can result in substantial economic losses in wheat production [51]. More recently, the soybean aphid, *Aphis glycines*, has continued to cause substantial economic costs in North America, with economic modelling suggesting that effective control measures could generate producer benefits of US$ 949 million to US$ 1.62 billion over a ten-year period compared with unmanaged infestations [52].

## 5. Aphid-Transmitted Viruses

Aphids are among the most effective vectors of plant viruses, transmitting over 275 virus ‘species’, which can drastically reduce crop quality and yield [1,5]. The efficiency of aphid-mediated virus transmission lies in their feeding behaviour, mobility, and the diversity of virus transmission modes classified as non-persistent, semi-persistent and persistent [1]. In non-persistent transmission, viruses are acquired and transmitted within seconds to minutes as aphids probe host plants [1]. This mode is typical of many potyviruses and cucumoviruses. With 176 recognized species, Potyvirus represents the largest and most comprehensively studied genus within the Potyviridae family, primarily due to its considerable economic significance [53]. Although most potyviruses exhibit a narrow host range, certain species are capable of infecting plant hosts across as many as 38 different plant families, leading to substantial yield losses in a variety of cultivated crops [54]. It is currently estimated that the majority of potyviruses are transmitted by more than 200 aphid species through a non-persistent, non-circulative mode of transmission [55]. The genus *Cucumovirus*, belonging to the family Bromoviridae, is widely distributed across numerous countries worldwide. Cucumber mosaic virus (CMV), the most prominent member of this genus, possesses an exceptionally broad host range, infecting more than 1200 plant species across 100 families. Transmission occurs through seeds, mechanical contact, and a variety of vectors. CMV is transmitted by aphids in a non-persistent manner, during which viral particles transiently associate with putative receptor molecules located in the aphid stylet and are subsequently released into the plant tissue during salivation [56]. Because transmission occurs during superficial probing, chemical insecticides are largely ineffective at preventing virus spread, as the insects often transmit the virus before the insecticide can take effect [57,58].

Semi-persistent viruses, such as closteroviruses, require a longer acquisition and inoculation time and can be retained for several hours [1]. This mode of transmission is modulated by the feeding behaviour of the vector as well as the plant‘s physiological response to viral infection, both of which can affect vector attraction and alter feeding dynamics [59]. Persistent viruses, especially luteoviruses and geminiviruses, undergo circulative or propagative cycles within the aphid, being retained for days or throughout the vector’s lifespan. For example, *M. persicae* is a key vector of Potato leafroll virus (PLRV), a luteovirus that causes substantial yield losses in potato-growing regions [60]. Infection by Turnip yellows virus (TuYV) induces metabolic alterations in the host plant, leading to a suppression of defense mechanisms and increased suitability as a feeding substrate, thereby enhancing transmission efficiency [61,62]. These viruses actively manipulate host plant traits to establish a more favorable environment for sustained aphid feeding and prolonged virus transmission [63].

As exemplified below, case studies underscore the global significance of aphid-transmitted viruses:

The global importance of aphid-transmitted viruses is illustrated by numerous regional case studies. In Europe, the bird cherry-oat aphid, *Rhopalosiphum padi*, and the grain aphid, *Sitobion avenae* are the principal vectors of BYDV in cereal crops, where the disease persists despite seed treatments and foliar insecticide applications owing to early aphid colonization and the development of insecticide resistance [64]. In the Midwestern USA, *A. glycines* transmits Soybean mosaic virus (SMV) and Soybean dwarf virus (SbDV), causing yield losses of up to 30%, while conventional insecticide-based management has shown limited efficacy, increasing reliance on virus-resistant cultivars [65]. In East and Central Africa, *A. fabae* serves as the primary vector of Bean common mosaic virus (BCMV) in common bean, where breeding for resistant cultivars has become a key management strategy following the limited success of insecticide-based control [66]. Likewise, *Aphis craccivora* and *M. persicae* are responsible for the efficient transmission of economically important plant viruses, including Groundnut rosette virus (GRV) in legumes and Turnip mosaic virus (TuMV) in brassica crops, with management further complicated by the high dispersal capacity, rapid reproduction and broad host range of both aphid species [67,68]. In Australia, *R. padi* is the predominant vector of yellow dwarf viruses (YDVs), with BYDV-PAV representing the most widespread species affecting wheat and grassland ecosystems [69,70].

Evidence that aphid vectoring within cash crops has significant economic impact is illustrated by the following examples:

The relative contribution of direct aphid feeding and virus transmission to crop losses varies considerably among host species. In cereal crops such as wheat and barley, direct phloem feeding can reduce plant vigour and yield; however, virus transmission generally accounts for the greater proportion of economic losses [48]. Earlier field studies estimated direct yield losses caused by aphid feeding alone at approximately 10–13% in wheat, although the magnitude varied substantially with aphid density and environmental conditions [71]. By contrast, aphid-transmitted Barley yellow dwarf viruses (BYDV/CYDV), primarily vectored by *R. padi* and *S. avenae*, can reduce yields by up to 80% in wheat and 64% in barley under controlled inoculation conditions [72] Furthermore, field experiments demonstrated that each 1% increase in BYDV incidence resulted in an approximately 0.9% (72 kg ha^−1^) reduction in grain yield, emphasizing the strong quantitative relationship between virus incidence and crop loss [72].

A similar pattern is observed in potato production, where the economic impact of aphids is driven predominantly by virus transmission rather than direct feeding damage. Potato virus Y (PVY), transmitted by multiple aphid species, was estimated to cause annual economic losses of approximately €187 million in seed potatoes and €91–96 million in ware potato production across the European Union between 2004 and 2017 [73]. Reported yield reductions associated with PVY range from approximately 9% to over 50%, depending on disease incidence and production system, with losses of 16–44% commonly reported across Europe, North America, Africa, and Asia [74,75].

In maize, the relative importance of direct feeding and virus transmission is more balanced. Heavy infestations of *Rhopalosiphum maidis* may directly reduce yields by as much as 90% through intensive phloem feeding and honeydew accumulation under outbreak conditions [76]. At the same time, this species vectors several economically important viruses, including Maize yellow dwarf virus (MDMV), and Sugarcane mosaic virus (SCMV), thereby amplifying production losses beyond those caused by feeding alone [77].

Aphid infestations in fruit orchards and plantations are responsible not only for direct feeding damage through phloem sap extraction, deformation of shoots and fruits, as well as honeydew production, but also for the efficient transmission of numerous economically devastating plant viruses [78]. Because perennial crops remain in production for decades, the cumulative impact of aphid-vectored viruses is often considerably greater than in annual cropping systems, resulting in long-term reductions in productivity, fruit quality, orchard longevity, and increased management costs [79]. Sustainable aphid management in perennial fruit production has become increasingly important as climate change, global trade, and expanding vector distributions facilitate the emergence and spread of aphid-borne viral diseases across major fruit-producing regions [80].

Research on citrus production has consistently identified Citrus tristeza virus (CTV) as one of the most destructive viral diseases worldwide, with *A. gossypii* and especially *Toxoptera citricida* recognized as its principal vectors responsible for the rapid spread of severe virus isolates [81,82].

Similar findings have been reported in pome fruits, where *Dysaphis plantaginea*, *Aphis pomi* and *Eriosoma lanigerum* cause substantial feeding damage, while aphid-mediated transmission of viruses such as ASGV, ASPV, ACLSV and ApMV further compromise tree vigour, fruit production and nursery quality [78,83,84].

Stone fruit research has demonstrated the pivotal role of *M. persicae* as both a highly polyphagous pest and one of the most efficient vectors of Plum pox virus (PPV), although additional aphid species also contribute to epidemiology depending on regional and climatic conditions [85,86].

Although nut crops have received less attention, recent studies have identified *Chromaphis juglandicola* and *Panaphis juglandis* as the dominant walnut aphids, capable of reducing photosynthetic activity and nut quality through prolonged feeding and honeydew production [87,88].

Likewise, investigations on berry crops have confirmed the importance of *Chaetosiphon fragaefolii* and *Amphorophora idaei* as key vectors of strawberry and raspberry viruses, highlighting the central role of aphids in virus epidemiology across perennial fruit production systems [89,90,91]. Beyond horticulture, *Cinara* spp. have also emerged as important forest pests whose populations may benefit from changing climatic conditions [92].

The quantified impacts resulting from such aphid infections in the aforementioned cash crops are shown in Table 2.

### 5.1. Case Studies of Successful and Unsuccessful Control of Aphids

Aphid management in agricultural systems has historically relied on both chemical and biological strategies, with their effectiveness varying by crop, aphid species, and management context.

In China, for example, seed dressings with the neonicotinoid imidacloprid significantly reduced populations of *S. avenae* and *R. padi* throughout an entire wheat growing season, lowering survival and population sizes by >70% relative to untreated plots. However, the effectiveness of this approach was highly species-dependent; thus, the cereal aphid *Metopolophium dirhodum* showed no significant reduction in survival in treated plants and sometimes increased in relative abundance [93]. In contrast, pyrethroid resistance and cross-resistance patterns have been detected in *R. padi* populations, with some showing very high resistance ratios in wheat aphid populations [94].

Long-term monitoring data from Europe further illustrates the emergence of chemical tolerance as a limit to traditional insecticide approaches. Populations of *M. persicae* and *A. gossypii* in certain regions have shown reduced sensitivity to imidacloprid over repeated use, indicating that selection pressure from frequent applications can erode efficacy over time and contribute to control failures [95]. Similarly in Greece, studies have shown that *M. persicae* has evolved medium-to-high resistance levels to organophosphates, carbamates, pyrethroids, and neonicotinoids over decades of use [96].

Several studies have evaluated alternative insecticides other than the currently favoured neonicotinoids. Hence, for example, applications of indoxacarb and elemental sulphur formulations used in okra fields not only produced high aphid reduction rates (>>90% for indoxacarb), but also caused significant declines in natural predator populations such as ladybird beetles, *Coccinella* spp. (Coleoptera: Coccinellidae) and lacewings (Neuroptera: Chrysopidae), illustrating a key trade-off between pest suppression and beneficial arthropod conservation [97].

Alternative chemicals such as flupyradifurone, a butenolide insecticide with systemic actions, have shown promising aphid control efficacy in vegetables and field crops in Germany, including against lettuce aphids *Nasonovia ribisnigri*, with rapid uptake and distribution in plant tissues and high mortality within days of use [98]. Similar results were found in Australia. Four *A. kondoi* populations were found to exhibit moderate (10–40-fold) resistance to organophosphates, carbamates, and pyrethroids, whereas no resistance was detected against flupyradifurone [99].

In greenhouse environments, rigorous comparisons of biological agent release programmes versus chemical pesticide treatments have shown that strategic releases of a predator, i.e., larvae of the green lacewing *Chrysoperla carnea* (Neuroptera: Chrysopidae), markedly reduced *M. persicae* populations by 60–65% in cucumber growing greenhouses across multiple seasons. In contrast, conventional pesticide treatments were often less effective and could lead to secondary pest increases (spider mites; Arachnida: Trombidiformes: Tetranychidae) due to disrupted predator communities [100].

Biological control approaches in controlled environments offer a contrasting example of success contingent on timing and release strategy. In greenhouse ornamentals, augmentative releases of predatory ladybirds significantly suppressed aphid populations when implemented early and at adequate densities [101]. Research on sugar beet crops has demonstrated that diverse natural enemy guilds significantly reduce aphid populations. Thus, ground-dwelling predators, such as carabid beetles (Coleoptera: Carabidae) and hymenopterous parasitoids, reduced aphid abundance (*A. fabae*, *M. persicae*) by ≥80% in field trials, with substantial parasitism detected early in the season [102].

Among the natural enemies of aphids, fungi belonging to the order Entomophthorales deserve particular attention. This is because they constitute one of the most important mortality factors regulating aphid populations under natural field conditions. Species such as *Pandora neoaphidis*, *Entomophthora planchoniana*, *Neozygites fresenii* and *Conidiobolus obscurus* frequently induce epizootics capable of rapidly suppressing aphid outbreaks, especially under conditions of high relative humidity and moderate temperatures [103,104], unlike commercially applied entomopathogenic fungi such as *Beauveria bassiana* or *Metarhizium anisopliae*, which primarily contribute to conservation biological control management schemes through naturally occurring infections that persist within agroecosystems and interact synergistically with predators and parasitoids. Consequently, farming practices that conserve these indigenous fungal communities, including reduced fungicide inputs, maintenance of habitat diversity and minimization of unnecessary pesticide applications, can substantially enhance the natural regulation of aphid populations and reduce reliance on chemical insecticides [104,105,106]. The most important aphid species as pests and vectors by crop types and viruses transmitted over the regions are presented in Table 3.

### 5.2. Impact on Non-Target Species

The use of broad-spectrum insecticides to control aphid populations often leads to significant unintended consequences on non-target species, particularly beneficial arthropods involved in natural pest regulation. These collateral impacts undermine ecological balance and can exacerbate pest problems through secondary pest outbreaks or resurgence. Predators such as ladybird beetles, lacewings, and syrphid flies (Diptera: Syrphidae) are essential biological control agents that prey on aphids across multiple cropping systems. Insecticide exposure, even at sub-lethal doses, impairs the behaviour, development, and fecundity of these predators [115].

For instance, exposure to imidacloprid has been shown to reduce oviposition and larval survival in *Hippodamia convergens* (Coleoptera: Coccinellidae), a common North American ladybird beetle. Several studies have documented the cytotoxic and behavioural effects of insecticides on non-target predatory insects. Spinosad, a neurotoxin derived from the actinobacterium *Saccharopolyspora spinosa*, has been shown to induce cytotoxic damage in the salivary glands of insects, while imidacloprid, permethrin, and spinosad demonstrate similar effects in the midgut of the predatory stinkbug, *Podisus nigrispinus* (Hemiptera: Pentatomidae) [116,117,118].

Other studies have shown that the predatory behaviour of the eleven-spot ladybird beetle *Coccinella undecimpunctata* is adversely affected by exposure to pymetrozine and pirimicarb [119]. Thiamethoxam (neonicotinoid) and diazinon (organophosphate) have been associated with feeding cessation and reduced mobility in the minute pirate bug, *Orius insidiosus* (Hemiptera: Anthocoridae), another predatory bug species [120]. Furthermore, organophosphates such as fenitrothion and chlorpyrifos elicit leg tremors and rostrum salivation in the stink bug, *Andrallus spinidens* (Hemiptera: Pentatomidae), indicating significant neurotoxic effects [121].

Parasitoid wasps, such as *Aphidius colemani* (Hymenoptera: Braconidae) and *Aphelinus abdominalis* (Hymenoptera: Aphelinidae), which are commonly used in greenhouse Integrated Pest Management (IPM) programmes, are highly susceptible to insecticide residues. Even minimal exposure to systemic or contact insecticides can reduce parasitism rates and host location ability [122]. A study showed that deltamethrin-treated surfaces significantly reduced host-seeking behaviour in *A. colemani* [123]. In another study, it was revealed that thiamethoxam was the most harmful active ingredient, with an average mortality rate of 53.7%, followed by sulfoxaflor (a neurotoxic sulfoximine compound) in the case of *A. colemani* [124].

Broad-spectrum insecticides frequently exert significant lethal and sub-lethal effects on aphid natural enemies, thereby compromising the effectiveness of biological control. Aphid parasitoids, particularly species of the genera *Aphidius*, *Diaeretiella*, *Praon* and *Lysiphlebus*, are highly susceptible to pyrethroids, organophosphates and neonicotinoids, which reduce adult longevity, parasitism efficiency, host-searching behaviour and emergence from mummified aphids [115,125,126].

Similar detrimental effects have been reported for the principal aphid predators, including ladybird beetles, hoverflies and green lacewings, with insecticide exposure reducing survival, foraging activity and predation efficiency, thereby disrupting natural enemy communities [124,127].

### 5.3. Effect of Climate Change on Phenological Shifts

Climate change has major influences on the biology, ecology, and population dynamics of aphids, leading to shifts in their phenology, geographic distribution, and interaction with host plants and natural enemies. Rising global temperatures, altered precipitation patterns, and increased frequency of extreme weather events directly affect aphid life cycles and indirectly modify agroecosystem stability [128].

Aphids are ectothermic organisms, and as such their development rates are highly sensitive to temperature variations. Increased temperatures generally accelerate aphid development, reduce generation time, and increase the number of generations per year [129]. For instance, the cereal aphid *S. avenae* has been shown to complete more generations per season in warmer years in northern China, leading to elevated population densities and increased damage [130]. However, the relationship between temperature and aphid performance is non-linear. Extremely high temperatures can reduce aphid survival and fecundity due to thermal stress [131,132,133,134].

Warming temperatures have led to earlier spring migrations and colonization of crops by aphids in temperate regions. Long-term monitoring in Europe reveals that aphid flight activity begins days to weeks earlier than historically recorded, as reported by in this research [133,134] and supported by phenological models [135]. This earlier presence aligns aphid feeding with more vulnerable crop developmental stages, exacerbating pest pressure. Moreover, climate-induced shifts in the timing of host plant phenology may cause mismatches with aphid natural enemies, disrupting biological control. For example, if aphids emerge earlier while parasitoids or predators remain synchronized to historical cues, the effectiveness of such natural regulation may decline [136,137]. On a positive note, acceleration of host plant phenology as a result of temperature increase and/or agronomic practices may contribute to limitation of aphid attack, particularly for species that feed on or within inflorescences [138,139]. Over 15 years of surveillance in the U.S. concerning the phenology of three aphid species (*A. glycines*, *R. maidis* and *R. padi*) revealed that changes in both temperature and precipitation patterns have to be considered in order to understand observed changes in aphid flight phenology [140]. Moreover, similar climate conditions may differentially impact different aphid species [140,141] either directly, due to species-specific tolerance ranges, or indirectly, through complex relationships with plants, predators and endosymbionts that are also influenced by climate [142]. Asynchronies between aphid and host plant phenology, as well as synchronies between aphids and their natural enemies, are important in identifying and developing effective pest management programmes [136].

### 5.4. Modeling and Predictive Tools

Dynamic models integrating climate variables and aphid biology are increasingly used to predict future aphid outbreaks under climate change scenarios [143]. For example, degree-day models calibrated for the cereal aphids, *S. avenae* and *R. padi*, effectively forecast population peaks and virus transmission risk, aiding in timely control interventions [144]. By CLIMEX modeling, host plant availability as a result of climate change has been identified as the primary driver of the distributional change of the *Sitobion* grain aphid complex [145].

For accurate predictions, irrigation practices were also considered, as they not only play a critical role in maintaining host plant availability, but can also directly influence aphid populations by altering the microclimate. According to CLIMEX, the species are expected to expand northward across North America, Europe, parts of Asia, and Oceania, while a decline in distribution is anticipated in South America, Africa, and Australia. In independent studies, using the Maximum Entropy (MaxEnt) model, a very similar change in range of expansion and contraction is predicted for the pea aphid, *A. pisum* [146], and the Russian wheat aphid, *D. noxia* [147].

Again, human activities emerge as the main predictors in shaping the aphid distribution. Development of algorithms and comparison of modelling tools is needed for establishing the most accurate predictions of species distribution models, which are based mostly on species-presence data widely available in digital databases [148]. This should be regarded as a dynamic process, as the current forecasts rely heavily on historical data; therefore, for assuring future prediction accuracy, field surveys are still required [146]. Of equal importance are the short-term predictions which take into account not only the broad-scale correlations among meteorological factors, but also regional air-mass patterns and specific microclimates, as local weather conditions play a significant role in predicting aphid migration [138].

Although CLIMEX and MaxEnt have become valuable tools for predicting future aphid distributions, their accuracy depends on model assumptions, environmental input variables, and the quality of species occurrence records. Both approaches rely heavily on historical observations and may underestimate future distributions if rapid evolutionary adaptation, changing agricultural practices, climate change, or novel host associations occur. Therefore, model outputs should be interpreted as decision-support tools rather than definitive forecasts and should be validated continuously using long-term field monitoring.

### 5.5. Challenges and Adaptation

The ongoing impacts of climate change pose considerable challenges for aphid management. Changes in pest phenology and distribution require adjustments in monitoring protocols, thresholds, and timing of control measures [149]. IPM programmes must incorporate climate-adaptive strategies, such as breeding climate-resilient crop varieties [150] and enhancing habitat complexity to support natural enemies [151]. Future farming can take advantage of the new emerging technologies [152]. Small unmanned aircrafts, i.e., drones, equipped with sensors can be employed in monitoring large areas of commercial crops with the aim of combatting direct physical pest damage as well as that induced by virus transmission [153]. Preliminary tests indicate that such drones can be efficient and cost effective in limiting aphid infestations, either by applying organic-certified insecticides or by releasing beneficial insects, i.e., antagonists such as predators and parasitoids [154].

## 6. Limitations of Biological Control and IPM Adoption

IPM and biological control are heralded as sustainable alternatives to chemical insecticides for aphid management. However, practical implementation faces numerous biological, socio-economic, and institutional barriers, limiting their widespread adoption and efficacy.

### 6.1. Biological Control Constraints

As mentioned above, biological control relies on the use of natural enemies including predators (ladybird beetles, lacewings, hoverflies), parasitoids (e.g., *Aphidius* spp.), and entomopathogenic fungi (e.g., *Beauveria bassiana*) [155]. While these agents can substantially suppress aphid populations under controlled conditions, as found in glasshouses, their performance in the field often falls short due to multiple factors: Environmental conditions: temperature, humidity, and microclimatic conditions critically affect natural enemy survival, development, and efficacy. For example, parasitoid wasps exhibit reduced parasitism rates under high temperatures or drought stress, which are becoming more frequent with climate change [156,157].

Factors that are crucial in the implementation of successful IPM strategies against aphid pests in the field include:

(i) Pesticide compatibility: As alluded to earlier, even selective insecticides used within IPM programmes can negatively affect beneficial arthropods. Sub-lethal doses impair their reproduction, longevity, and predation/parasitism behaviour, reducing their effectiveness [115]. For instance, neonicotinoid seed treatments in soybean fields have been implicated in reduced parasitism of soybean aphid by *A. colemani* [158,159].
(ii) Dispersal and Habitat Fragmentation: Many biological control agents require habitat complexity and refuges to sustain populations. Intensive monoculture cropping systems and habitat destruction reduce the availability of overwintering sites and alternative prey, limiting natural enemy persistence [160]. In Brazil, low landscape complexity has been associated with poor natural enemy abundance and efficacy against *A glycines* [161].
(iii) Host Specificity and Adaptation: Some parasitoids and predators display narrow host ranges, limiting their impact across diverse aphid species or biotypes [162,163]. Additionally, aphids may develop behavioural defenses or resistance to biological agents, such as increased dropping behaviour [164].

Biological control represents a key component of sustainable pest management and offers a promising alternative or complement to chemical strategies for managing insect vectors of phytoplasmas.

### 6.2. IPM Adoption Barriers

Although IPM integrates multiple control tactics (biological, cultural, mechanical, and chemical), its adoption is hampered by:

(i) Knowledge and training gaps: Many smallholders and commercial farmers lack sufficient training in IPM principles, aphid identification, and threshold-based interventions. This leads to overuse of prophylactic insecticides, which undermines IPM benefits [165]. In some regions, pest control services are underfunded or inaccessible, limiting knowledge transfer [166].
(ii) Economic and market pressures: The immediate cost-effectiveness and ease of chemical insecticides often outweigh the perceived benefits of IPM. Additionally, the lack of economic thresholds for specific crops and regions complicates decision-making. For example, in the USA, soybean aphid IPM adoption has been found to vary widely depending on crop prices and insecticide costs [167].
(iii) Availability and quality of biocontrol agents: Mass production and distribution of effective biocontrol agents remain challenging. Quality control issues, short shelf life, and inconsistent field performance limit grower confidence [168]. In greenhouses, parasitoid releases have demonstrated success, but scalability to open-field crops is less certain [169].
(iv) Regulatory and policy frameworks: In many countries, regulations favour chemical pesticide registration and use, with limited incentives for biocontrol adoption. Policy gaps include insufficient subsidies, lack of support for organic or sustainable farming practices, and weak enforcement of pesticide reduction targets [166,170].

### 6.3. Social and Cultural Factors

Farmers’ attitudes, traditions, and risk perceptions influence IPM uptake. Studies show that farmers accustomed to chemical reliance may be reluctant to change without visible and immediate pest suppression results. Social learning, peer influence, and participatory approaches can enhance adoption but require long-term commitment [171]. Below we cite a few case studies to illustrate this point:

Regional case studies further demonstrate that the implementation of IPM for aphid control remains highly variable. In Brazil, despite widespread awareness of IPM principles for soybean production, insecticide applications remain common because of market demands and the absence of well-defined economic thresholds, often resulting in the disruption of natural enemy communities [161,165]. Across Europe, IPM adoption differs substantially among countries, with greater implementation in regions supported by favourable policy frameworks, effective extension services, and biological control programmes [169]. Similarly, in the USA, integrating biological control into large-scale production systems remains challenging owing to the prevalence of monoculture and intensive insecticide use, although several states have promoted IPM through cooperative extension programmes and grower incentive schemes [167].

### 6.4. Future Directions

To overcome these limitations, multidisciplinary approaches combining research, education, policy, and farmer engagement are required. Advances in molecular tools can improve natural enemy identification and monitoring [172]. Development of habitat management practices such as flower strips and cover crops can enhance natural enemy populations [162]. Policies should encourage reduced pesticide use and support biocontrol commercialization.

## 7. Emerging Resistance to Biological Insecticides

### 7.1. Entomopathogenic Fungi and Resistance Development

Entomopathogenic fungi such as *B. bassiana*, *M. anisopliae*, and *Lecanicillium muscarium* are widely used against aphids. Their mode of action involves spore attachment, germination, cuticle penetration, and internal proliferation leading to host death [173].

Repeated exposure of aphid populations to entomopathogenic fungal biopesticides has revealed several mechanisms that may reduce treatment efficacy. One of the earliest responses is behavioural avoidance, whereby aphids reduce contact with infective spores through increased grooming, dropping from host plants, or avoiding treated surfaces. Such behavioral adaptations have been documented in *M. persicae*, contributing to reduced susceptibility under greenhouse conditions [174,175]. Structural defenses also contribute to fungal resistance. Alterations in cuticle thickness or composition may impair spore adhesion and penetration, while increased cuticular hydrocarbon production and melanization provide additional physical and chemical barriers against fungal invasion [176,177]. In *A.gossypii*, activation of ecdysone-related transcripts during the early stages of *B. bassiana* infection promotes molting, facilitating the shedding of infected cuticle and thereby limiting fungal establishment [177]. In addition, aphids can mount enhanced innate immune responses, including both cellular and humoral defenses, with resistant populations exhibiting increased expression of immune-related genes following fungal infection [178]. Finally, sub-lethal exposure to fungal biopesticides may induce hormetic effects, whereby low doses increase aphid survival, fecundity, and reproductive performance across generations. This phenomenon has been demonstrated in specific asexual lineages of *M. persicae* exposed to low concentrations of *B. bassiana* spores and appears to be modulated by the facultative endosymbiont *Regiella viridis*, thereby highlighting the complex interactions among fungal pathogens, aphid physiology, and microbial symbionts [179].

### 7.2. Bacterial and Viral Biopesticides

Biopesticides based on bacteria, such as *Bacillus thuringiensis* (Bt), can alter the probing and feeding behaviour of aphids immediately after application, but have been found to have limited success in terms of long-term protection against aphids due to their sap-feeding behaviour and the actions of bacterial endosymbionts, which can alleviate the effect [180,181]. However, attempts to engineer Bt toxins and other bacterial agents have met with concerns relating to the development of resistance to these products [182,183].

(i) Symbiont-mediated resistance: Aphids harbour facultative bacterial endosymbionts (e.g., *H. defensa*, *R. insecticola*) that can confer protection against natural enemies, including fungal pathogens, hymenopterous parasitoids and even viruses [184,185,186]. These symbionts influence the efficacy of microbial biocontrol agents by producing toxins or modulating aphid immunity, complicating biopesticide application [187]. While viral biopesticides are common for lepidopteran pests, their application against aphids is limited. Nevertheless, there are emerging studies exploring aphid-specific viruses as biocontrol agents, with caution advised due to possible aphid tolerance or the evolution of resistance [188,189,190].
(ii) Host plant modulating the effect of biological insecticides: On alfalfa, pea aphids, *A. pisum* produced fewer total offspring but more winged morphs under *Staphylococcus aureus* and *B. bassiana* exposure, whereas on broad beans they produced more offspring but fewer winged morphs after pathogen infection [191].

### 7.3. Challenges in Managing Resistance

In addition to physiological and behavioural adaptations, evidence suggests that susceptibility to fungal biopesticides may also have a heritable genetic basis. Although genetic resistance has been investigated less extensively than resistance to synthetic insecticides, selection experiments with *B. bassiana* have demonstrated shifts in aphid susceptibility over successive generations, indicating that genetic factors may contribute to reduced sensitivity [192]. Finally, the performance of fungal biopesticides is strongly influenced by formulation quality, environmental conditions, and application timing. Under suboptimal conditions, aphids may receive sub-lethal doses that reduce control efficacy while increasing selection pressure for more tolerant populations, thereby potentially accelerating the evolution of reduced susceptibility [179,193,194,195].

### 7.4. Strengthening Research and Innovation Funding

Continued investment in multidisciplinary research is critical to develop innovative aphid management tools [196]. Governments and international organizations should prioritize sustained investment in research aimed at improving the long-term sustainability of aphid management. Key priorities include genomic and molecular studies to elucidate resistance mechanisms and identify novel targets for control, together with the development of alternative management approaches such as next-generation biopesticides, RNA interference (RNAi)-based technologies, and aphid-resistant crop varieties [4,17,197,198]. Equal emphasis should be placed on the development of region-specific IPM strategies that combine chemical, biological, and cultural control measures adapted to local agro-ecological conditions [115,161]. Improving predictions of climate change impacts will require additional laboratory and field studies to quantify aphid responses under realistic future climate scenarios [146]. To maximize scientific and practical impact, funding programmes should also encourage multidisciplinary research networks involving universities, governmental agencies, the private sector, and farming organizations, thereby facilitating knowledge exchange, technology transfer, and the implementation of sustainable pest management practices.

### 7.5. Enhancing Regulatory Frameworks

Regulatory agencies must update pesticide registration and approval procedures to reflect contemporary ecological and resistance challenges. Strengthening regulatory frameworks is essential to ensure the sustainable management of aphid populations while minimizing environmental impacts. Environmental risk assessments should place greater emphasis on evaluating the effects of both chemical pesticides and biopesticides on non-target organisms, beneficial insects, and ecosystem services [115,199]. In parallel, resistance management plans should become a mandatory component of pesticide registration, requiring manufacturers to assess resistance risks and propose appropriate mitigation strategies before market approval [4].

Despite broad recognition of its benefits, the adoption of IPM remains inconsistent, particularly in developing countries [165]. Expanding farmer education and agricultural extension programmes is essential to improve pest monitoring, economic threshold-based decision making, and the implementation of biological control strategies [161]. Equally important is improving access to affordable biological control agents, pest monitoring technologies (e.g., pheromone traps and remote sensing), and resistant crop varieties [200]. Embedding IPM principles within national agricultural policies and extension curricula would strengthen institutional support and facilitate the large-scale implementation of sustainable aphid management strategies.

### 7.6. Implementing Resistance Monitoring and Surveillance Programmes

Early detection and continuous monitoring of insecticide resistance are essential for preserving the efficacy of existing aphid management strategies. Establishing standardized resistance monitoring networks at regional and national levels would enable the systematic collection of susceptibility data, supporting timely management recommendations and evidence-based resistance mitigation strategies [4,43]. The integration of molecular diagnostic tools into surveillance programmes would also further improve detection sensitivity by identifying resistance-associated markers before phenotypic control failures become evident. Equally important is the development of data-sharing platforms and decision-support systems that facilitate communication among researchers, policymakers, extension specialists, and growers, thereby enabling coordinated responses to emerging resistance threats [201].

### 7.7. Addressing Climate Change Adaptation

Climate change adaptation policies should anticipate shifts in aphid distribution, phenology, and population dynamics that are likely to alter pest pressure and virus transmission. Integrating pest risk models and forecasting systems into national agricultural planning would improve the timing of planting, monitoring, and control interventions under changing climatic conditions [93,133]. At the same time, promoting climate-resilient crop varieties and greater cropping diversification could reduce crop vulnerability to aphid infestations and virus outbreaks associated with environmental stress [202]. Broader adoption of agro-ecological practices, including the conservation of natural enemy habitats and increased landscape heterogeneity, would further strengthen ecosystem resilience and enhance the biological regulation of aphid populations [203].

### 7.8. Fostering International Cooperation

Given the transboundary nature of aphid migration and the widespread dissemination of aphid-transmitted viruses, international coordination is essential for effective resistance management and sustainable pest control. Regional cooperation frameworks can facilitate the harmonization of surveillance programmes, research initiatives, and regulatory policies across countries [6]. In this context, multilateral organizations such as the Food and Agriculture Organization (FAO) and the Consultative Group on International Agricultural Research (CGIAR) are well positioned to coordinate international collaboration, promote knowledge transfer, and strengthen global preparedness against aphid-associated threats.

## 8. Future of Aphid Control Success and Costs

The success of aphid management over the next few years will likely depend on the balance between technological innovation and emerging biological and environmental challenges. Continued adoption of IPM, together with advances in biological control, precision agriculture, and predictive modelling, is expected to improve the effectiveness of sustainable aphid management while reducing reliance on synthetic insecticides [115,193].

Digital agriculture is also expected to play an increasingly important role in aphid surveillance and decision support. Artificial intelligence (AI)-based forecasting systems that integrate weather data, field history, and pest risk models may improve the timing of control interventions while reducing unnecessary insecticide applications [133,199]. Advances in remote sensing, unmanned aerial vehicles (UAVs), and multispectral satellite imagery enable the early detection of crop stress associated with aphid infestations, allowing interventions before visible symptoms develop. Emerging deep-learning and computer vision algorithms further enhance monitoring by automatically identifying aphid colonies and supporting precision pest management [93].

Despite these advances, several factors are likely to limit future control success. Continued reliance on chemical insecticides is expected to accelerate the geographic expansion of resistance in major pest species, including *M. persicae* and *A. gossypii*, particularly in low- and middle-income countries where access to alternative management strategies remains limited [4,43]. Climate change is expected to exacerbate these challenges by promoting earlier aphid phenology, increasing the number of generations per growing season, and elevating the risk of aphid-transmitted virus epidemics, especially in temperate and subtropical regions [93,133]. The global adoption of sustainable IPM practices is likely to remain uneven because of limited infrastructure, insufficient economic incentives, and inadequate technology transfer, particularly in parts of Sub-Saharan Africa, Southeast Asia, and Latin America [161,165].

### Estimated Cost of Aphid Control by 2030

Future economic estimates presented in this review originate from previously published economic models and should be interpreted as scenario-based projections rather than precise forecasts. These estimates depend on assumptions regarding future climate conditions, insecticide resistance development, adoption of integrated pest management, technological advances, and agricultural policy. Consequently, considerable uncertainty remains regarding absolute future economic costs.

Nevertheless, the economic costs associated with aphid management are expected to increase over the coming years as insecticide resistance, climate change, and expanding virus incidence place additional pressure on crop protection systems. Direct management costs are likely to rise because resistance will necessitate more frequent insecticide applications and the adoption of newer, often more expensive, active ingredients. Consequently, global expenditure on aphid control has been projected to increase from approximately US$3.5 billion in 2020 to US$5–6 billion by 2030 [204,205].

Beyond direct control expenses, aphid management also generates substantial indirect and external costs. Continued reliance on broad-spectrum insecticides is expected to increase environmental costs associated with adverse effects on pollinators, natural enemies, and other ecosystem services, with global external costs projected to exceed US$2 billion annually by 2030 [115]. Without wider deployment of virus-resistant cultivars, effective vector management, and improved disease surveillance, annual crop losses attributable to viral diseases could reach an estimated US$8–12 billion by 2030, particularly in developing regions where access to quality seed systems and early detection remains limited [206,207].

The prediction of future control, its success and costs, and also the key factors are presented in Table 4.

## 9. Discussion

The principal contribution of this review is the integration of biological, ecological, technological, and socioeconomic drivers into a unified framework explaining why aphid management is becoming increasingly difficult worldwide. This synthesis provides a basis for future multidisciplinary research and sustainable management policies.

This being said, as here related, aphid pest control remains a critical and complex challenge in global agriculture due to the unique biological characteristics of aphids, especially including their ability to rapidly develop resistance to chemical controls, and their role as efficient vectors of plant viruses. The interplay of ecological, evolutionary, and socio-economic factors complicates management efforts, demanding integrated and adaptive approaches. The rapid reproductive capacity and genetic plasticity of aphid populations enable them to quickly adapt to changing environmental conditions and chemical pressures, as extensively documented for *M. persicae* and *A. gossypii* populations worldwide. This adaptability contributes significantly to the evolution of insecticide resistance, which has undermined the efficacy of conventional chemical controls and highlighted the urgent need for resistance management strategies [47,208]. Moreover, the role of aphids as vectors of a broad range of plant viruses exacerbates crop losses and complicates pest management, as many viruses are transmitted rapidly and before insecticide treatments can take effect. This phenomenon necessitates a shift toward virus-resistant cultivars, vector behaviour monitoring, and diversification of control measures. Such ecological disruption often leads to secondary pest outbreaks and reduces the sustainability of pest management programmes. The accelerating pace of climate change further complicates aphid management by altering pest phenology, geographic distribution, and interaction with host plants and natural enemies [133]. These shifts require dynamic pest surveillance, risk assessment, and adaptive management plans to maintain effective control, as ecological and evolutionary forces are always present and hence likely to impact field populations.

Despite the proven benefits of IPM, widespread adoption remains constrained by socio-economic barriers, limited extension services, and insufficient policy support, particularly in developing regions [161,165]. Efforts to promote IPM must be paired with enhanced farmer education, economic incentives, and improved access to biological control agents and monitoring technologies.

Overall, the complexity of aphid pest control requires integrated and ecologically sound management strategies supported by robust policy frameworks. Enhanced collaboration between researchers, policymakers, farmers, and industry stakeholders is essential to develop sustainable solutions that address both immediate pest control needs and long-term agro-ecosystem resilience. Future research should, we suggest, focus on the development of innovative control methods, including biotechnological approaches such as RNA interference and host plant resistance, alongside improved resistance monitoring and climate-adaptive strategies. In this light, policymakers should, we propose, based on these emerging serious concerns, create enabling environments for IPM adoption, incentivize sustainable practices, and harmonize regulatory frameworks to address the global nature of aphid pest threats. Only through such concerted, multidisciplinary efforts can the persistent challenges posed by aphids be effectively managed, ensuring food security and environmental sustainability in an increasingly uncertain world.

By 2030, aphid control success will be regionally variable. While high-income countries with strong regulatory frameworks and IPM support will see moderate improvements, most developing countries face increased costs and worsening resistance. Key priorities include:

- Investment in resistant cultivars and virus diagnostics
- Support for farmer education and IPM infrastructure
- Research into RNAi and microbial control agents
- International collaboration for resistance monitoring

If globally coordinated efforts are scaled up, the burden of aphid pests could be substantially reduced without increasing environmental costs. In the absence of such efforts, we may well see increasing pesticide dependency, reduced biodiversity, and rising food security risks.

## 10. Materials and Methods

This review was prepared following a structured narrative review approach. Scientific publications were searched using the Web of Science Core Collection (Clarivate, Philadelphia, PA, USA), Scopus (Elsevier, Amsterdam, The Netherlands), PubMed (National Center for Biotechnology Information, National Library of Medicine, Bethesda, MD, USA), and Google Scholar (Google LLC, Mountain View, CA, USA) databases. Literature published primarily between 2000 and 2025 was considered, although earlier classical publications describing aphid biology and insecticide resistance were included where relevant.

Searches were conducted using combinations of keywords including “aphid management”, “insecticide resistance”, “biological control”, “virus transmission”, “climate change”, “integrated pest management”, “forecasting models”, “RNA interference”, and “biological insecticides”. Only peer-reviewed English-language publications were considered.

The collected literature was organized into thematic categories including aphid biology, resistance mechanisms, economic impacts, virus transmission, biological control, climate change, modelling approaches, and future management strategies. Representative case studies from different geographical regions were selected to illustrate global similarities and regional differences in aphid management.

## Figures and Tables

**Table 1:** Trends in aphid-related costs & losses over time.

Region/Crop System	Time/Period	Yield Losses/Economic Impacts	Control Costs/Notes
Russian Wheat Aphid, U.S.	Late 1980s	Peak ~$274 M/year losses (yield + control)	Declined by early 1990s (<$10 M) as management improved
Cereal Aphids, UK/Britain	Late 1980s	Potential losses £70 M–£120 M (yield + viruses)	Variable pesticide and monitoring costs; economic thresholds guide treatments
Cereal Aphids (field monitoring)	1988 review	Overall impact range £2 M–£24 M per year based on outbreak severity	Timely scouting & targeted control reduce costs
Soybean Aphid, U.S.	1995–present (analysis to ~2008)	Long-term modelling: ~$949 M–$1.623 B producer benefits from control over ten years	Costs depend on insecticide prices and use of resistant cultivars
Cereal Aphids (direct yield)	Various regions	Direct yield loss 20–40%; virus pulled losses even higher	Aphid thresholds important for cost-effective spraying
Virus Transmission (e.g., Potato Leafroll Virus)	Global estimates	Virus vectored by aphids associated >50% loss in individual plants; global yield loss estimates ~20 M tons	Control focuses on integrated vector suppression

**Table 2:** Quantified impacts of virus damage and losses by aphids over time.

Crop/System	Direct Aphid Feeding Loss	Virus-Related Loss	Time/Scale	Reference Highlights
Wheat/Barley	Moderate (~8–16% in some cereals)	Severe (~up to 80% yield drop with BYDV)	Field trials over multiple seasons	BYDV up to ~80% loss in wheat/barley vs. moderate direct feeding losses
Potato (PVY)	Lower relative to virus impacts	~9.4–53% yield loss per field; EU annual losses ~€187 M seed + €96 M ware	2004–2017 multi-year data	PVY losses quantified in EU potato sector
Maize	Up to ~90% in heavy infestations	Multiple viruses transmitted by *R. maidis* contribute additional losses	Field observations	Direct maize yield drop quantified; viruses complicate losses
General cereals (historical)	~10–13% direct losses in wheat	BYDV often larger and widespread	Literature surveys	Direct vs. virus damage comparison across cereals

**Table 3:** The most important aphid species as pests and vectors by crop types and viruses transmitted over the regions.

Aphid Species (Hemiptera: Aphididae)	Common Name	Principal Crop Hosts	Major Diseases Vectored (Virus/Pathogen)	Main Affected Regions	Key References
*M. persicae*	Green peach aphid	Potato, tobacco, peach, pepper, tomato, oilseed rape	Potato virus Y (PVY), Potato leafroll virus (PLRV), Tobacco etch virus (TEV), Cucumber mosaic virus (CMV)	Global (Europe, Asia, Africa, Americas, Oceania)	[106]
*A. gossypii*	Cotton aphid	Cotton, cucurbits, citrus, okra, pepper	Cotton leaf curl virus complex (CLCuV), CMV, Watermelon mosaic virus (WMV)	Tropical and subtropical regions worldwide	[107]
*R. maidis*	Corn leaf aphid	Maize, sorghum, barley	Maize dwarf mosaic virus (MDMV), Sugarcane mosaic virus (SCMV), Barley yellow dwarf virus (BYDV)	Americas, Africa, Asia, Europe	[26,107,108]
*S. avenae*	English grain aphid	Wheat, barley, oats	Barley yellow dwarf virus (BYDV)	Europe, Asia, North America	[48,107,109]
*R. padi*	Bird cherry–oat aphid	Wheat, barley, oats, maize	BYDV, Cereal yellow dwarf virus (CYDV)	Temperate regions worldwide	[48]
*A. craccivora*	Cowpea aphid	Legumes (cowpea, groundnut, alfalfa)	Groundnut rosette virus complex, Bean common mosaic virus (BCMV)	Africa, South Asia, Europe. North America	[110,111,112]
*B. brassicae*	Cabbage aphid	Brassica crops (cabbage, cauliflower, mustard)	Turnip mosaic virus (TuMV), Cauliflower mosaic virus (CaMV)	Europe, Asia, Africa	[2,111]
*L. erysimi*	Mustard aphid	Mustard, rapeseed, cabbage	TuMV, CaMV	South Asia, East Asia	[113]
*M. euphorbiae*	Potato aphid	Potato, tomato, lettuce	PVY, CMV, Lettuce mosaic virus (LMV)	Americas, Europe	[2,109,114]
*T. citricida*	Brown citrus aphid	Citrus species	Citrus tristeza virus (CTV)	Asia, South America, Caribbean, Africa	[109]

**Table 4:** Prediction of aphid control success by regions.

Region	Control Success	Cost Trend	Key Factors
EU	Moderate–High	Slightly decreasing due to IPM	Strong policy and farmer training
USA/Canada	Moderate	Stable to slightly rising	Resistance pressures, improved tech
Latin America	Low–Moderate	Rising	Low IPM adoption, climate impacts
Africa	Low	Rising sharply	Weak extension, virus epidemics
Asia	Mixed	Rising	High pesticide use, urbanization pressures

## Data Availability

No new data were created or analyzed in this study. Data sharing is not applicable to this article.
